# Insight into the Reversible Hydrogen Storage of Titanium-Decorated Boron-Doped C_20_ Fullerene: A Theoretical Prediction

**DOI:** 10.3390/molecules29194728

**Published:** 2024-10-06

**Authors:** Zhiliang Chai, Lili Liu, Congcong Liang, Yan Liu, Qiang Wang

**Affiliations:** 1 College of Materials Science and Engineering, Taiyuan University of Technology, Taiyuan 030024, China; chaizhiliang1241@link.tyut.edu.cn; 2 School of Semiconductor and Physics, North University of China, Taiyuan 030051, China; 3 State Key Laboratory of Coal Conversion, Institute of Coal Chemistry, Chinese Academy of Sciences, Taiyuan 030001, China; liangcongcong0803@163.com (C.L.); liuyan@sxicc.ac.cn (Y.L.)

**Keywords:** hydrogen storage capacity, fullerene, Ti-decorated, density function calculation

## Abstract

Hydrogen storage has been a bottleneck factor for the application of hydrogen energy. Hydrogen storage capacity for titanium-decorated boron-doped C_20_ fullerenes has been investigated using the density functional theory. Different boron-doped C_20_ fullerene absorbents are examined to avoid titanium atom clustering. According to our research, with three carbon atoms in the pentagonal ring replaced by boron atoms, the binding interaction between the Ti atom and C_20_ fullerene is stronger than the cohesive energy of titanium. The calculated results revealed that one Ti atom can reversibly adsorb four H_2_ molecules with an average adsorption energy of −1.52 eV and an average desorption temperature of 522.5 K. The stability of the best absorbent structure with a gravimetric density of 4.68 wt% has been confirmed by ab initio molecular dynamics simulations. These findings suggest that titanium-decorated boron-doped C_20_ fullerenes could be considered as a potential candidate for hydrogen storage devices.

## 1. Introduction

Hydrogen energy is the key to a sustainable future because of its effectiveness in conserving energy and reducing emissions [1,2,3]. Solid-state hydrogen storage offers the highest safety and hydrogen storage density, as the adsorption energy of H_2_ fluctuates between the range of −0.2 eV to −0.7 eV [4,5,6]. Apart from that, the host molecules should hold at least 6.5 weight percentages (also known as wt%) of hydrogen, given by the guidelines of the United States Department of Energy (US-DOE) [7]. 

In recent years, various hydrogen storage materials have emerged, such as metastable alloys [8], magnesium hydride [9,10,11], zeolites [12], and metal organic framework materials [13]. Several metal alloys are stable after hydrogen adsorption, but the gravimetric weight of metal hydrides is often lower than the criteria set by the US-DOE [14]. A group of magnesium-based metal hydrides showed great theoretical performance with a hydrogen capacity of up to 7.6 wt% for reversible applications [8,15]. However, the experimental dehydrogenation enthalpy of magnesium is high due to its higher working temperature [16]. It needs a high temperature to discharge hydrogen molecules, and the kinetics of hydrogen adsorption and desorption are too slow for commercial use, which are also inevitable issues. It is difficult for zeolitic materials to capture enough amount of hydrogen molecules, as a result of which the hydrogen storage capacity fails to meet the expected value for practical applications [17]. MOF-210 has been holding the record for the highest H_2_ capacity of 17.6 wt% but at an extremely low temperature of 77.15 K and a high pressure of 8000 kPa, making it impractical for civil applications [18]. The ultimate technical aim of superior hydrogen storage materials requires a system gravimetric capacity of 6.5 wt%, as set by the US-DOE.

Under such a background, carbon-based nanomaterials stepped into the horizons of the scientific community and have shown promising results as hydrogen storage materials [19,20,21,22,23], like graphene [24,25], graphdiynes [26], carbon nanotubes [27,28], and fullerenes [29]. Studies have clearly demonstrated that the connection between hydrogen molecules and undoped carbon nanomaterials is very weak, and the reason for this phenomenon is that the van der Waals forces between the substrates and gaseous molecules are relatively weak [30,31]. Metal atom decoration on these carbon nanomaterials has been proven to be an efficient approach for hydrogen adsorption. A total of 2.53 wt% of gravimetric hydrogen storage capacity is observed for dual osmium-decorated SWCNTs in work by Verma et al. [32]. Hydrogen storage capabilities of metal-decorated graphene systems were predicted using DFT first-principle calculations, suggesting that an applied strain can stabilize supported metal atoms and increase the gravimetric density of hydrogen storage [33].

Recently, Dai et al. have reported on the hydrogen storage capacity of fullerene family molecules (C_56_, C_60_, and C_70_) by grand canonical Monte Carlo simulations [34]. Paul et al. studied yttrium-decorated C_30_ as a potential hydrogen storage material, where Y atoms adsorbing seven H_2_ molecules are observed [35]. Porous fullerene substituted by B atoms and doped with Ti atoms has been reported to have high hydrogen capacity [36]. Mahamiya et al. studied hydrogen adsorption in yttrium-doped C_24_ fullerene [37]. They have reported that with one Y atom doped, C_24_ fullerene can reversibly adsorb six H_2_ and reach an average desorption temperature of 477 K. Huang et al. performed a DFT study about the hydrogen storage capacity of Ti-decorated carbon atomic chain-terminated C_20_-4C_5_ and boron–nitrogen chain-terminated C_20_-4B_3_N_2_, which are good candidates for hydrogen storage [38]. Muniyandi et al. constructed a series of nanocages using C_20_ and B_12_N_12_ to adsorb beryllium hydride clusters and beryllium hydride molecules [39]. Ammar et al. took Ti-deposited C_20_ and Si_20_ as hydrogen storage materials [40]. Kareem et al. showed that adsorption is an endothermic process for C_20_ fullerene and an exothermic process for C_20_-_n_Ti_n_ heterofullerenes [41]. All the above studies focused on how to improve the hydrogen adsorption ability of fullerenes by decorating atoms. The transition metal Ti has been used to modify C_60_ fullerene to improve the hydrogen storage capacity [42]. It is well known that the adsorption energy of hydrogen on host materials could be improved by replacing C with boron atoms [43,44]. There are only a few studies on hydrogen storage of Ti-doped C_20_. Parkar et al. did a comprehensive study on the hydrogen storage properties of Ti-doped C_20_ nanocages [45]. It is inspiring to research the hydrogen storage capability of titanium-decorated boron-doped C_20_ fullerenes.

In this research, the hydrogen storage capability of titanium-decorated boron-doped C_20_ fullerenes has been investigated by density theory simulations. Their structural stability and hydrogen gravimetric weight were checked. A thermodynamic analysis of the system under different serving conditions was performed. Density of states and a Bader charge analysis of the host both with and without hydrogen molecules adsorbed were carried out. These theoretical simulations could inspire experimentalists to target synthesize a titanium-decorated boron-doped C_20_ fullerene system as a hydrogen storage material. 

## 2. Computational Details

Density functional theory (DFT) calculations and an ab initio molecular dynamics simulation (AIMD) were carried out by the Vienna ab initio simulation package (VASP) [46]. The electron–ion interaction was described by the projector augmented wave (PAW) method [47]. The generalized gradient approximation (GGA) was used for the exchange-correlation energy [48]. Spin polarization and dipole correction were considered in all calculations. Additionally, van der Waals interactions were accounted for through the application of the DFT-D2 method with Becke–Johnson damping [49,50]. The cutoff energy for the plane wave basis was set to 450 eV. All atoms are allowed to relax during structural optimization, and the cell shape and cell volume are not allowed to change. Geometry optimization was achieved until the energy and force were less than 10^−5^ eV and 0.02 eV/Å, respectively. The vacuum of 30 Å was used to avoid the interactions between the periodically repeating slabs. The *k*-point with a (1 × 1 × 1) mesh was sampled by the Monkhorst–Pack procedure. Ab initio molecular dynamics simulations were performed for a B123 model in microcanonical (NVE) and canonical (NVT) ensembles for five picoseconds of time duration, with a time step of one femtosecond.

The stability of the metal is ascertained through the binding energy ($E_{b}$), which is calculated as follows:

(1) $$\begin{array}{c} E_{b}=E_{C_{20}+metal}-E_{metal}-E_{C_{20}} \end{array}$$

where $E_{C_{20}}$, $E_{metal}$, and $E_{C_{20}+metal}$ are the energies of C_20,_, metal atom and metal atom-decorated C_20_, respectively. According to the equation, a negative binding energy suggests that the metal atom can be attached to C_20_.

To describe the adsorption strength between the hydrogen molecules and the absorbent structure, the adsorption energy ($E_{ad}$) is calculated as follows:

(2) $$\begin{array}{c} E_{ad}=\left(E_{system}-E_{metal}-n\times E_{H_{2}}\right)/n \end{array}$$

where $E_{H_{2}}$ and $E_{system}$ represent the energy of an isolated hydrogen molecule and the total energy of the combined hydrogen–metal C_20_ system, respectively, and n denotes the number of hydrogen molecules that have been adsorbed. 

The consecutive adsorption energy ($E_{cad}$) is calculated as follows:

(3) $$\begin{array}{c} E_{cad}=E_{n-system}-E_{n-1-system}-E_{H_{2}} \end{array}$$

where $E_{n-system}$ and $E_{n-1-system}$ represent the total energies of the systems, with n and n−1 being the numbers of hydrogen molecules adsorbed on each metal atom, respectively.

## 3. Results and Discussion

### 3.1. Absorbent Structure

**Figure 1:**
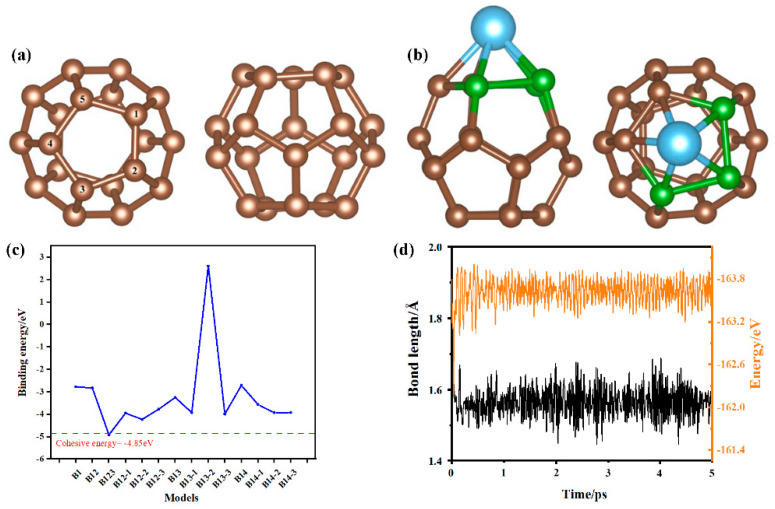
(**a**) Top and side views of C_20_. (**b**) Top and side views of the adsorbent B123 model. (**c**) Binding energy of different amounts of boron doping, where brown, green and blue represent carbon, boron and titanium atoms respectively. (**d**) Changes in energy and bond length in the ab initio molecular dynamics simulation (300 K, 5 ps) of the B123 model.

The optimized structure of C_20_ fullerene is shown in Figure 1a. C_20_ has a three-layer structure, where the top and the bottom layers are symmetrically connected to the middle layer. The five top carbon atoms in the pentagonal ring are labeled. Firstly, we calculated the adsorption energy between the bare C_20_ and the H_2_ molecule (−0.04 eV), indicating that pure C_20_ is unsuitable as a carrier for hydrogen [51]. It has been reported that boron atom doping can enhance the binding strength of metal atoms and carbon-based materials [52,53]. To determine how many and which carbon atoms should be replaced with boron atoms for better stability and hydrogen adsorption ability, we established several different models. The models are named after the doping position of the boron atoms. For example, to construct a model named “B12-1”, we first replaced carbon atom numbers 1, 2, and carbon atom number 1 on the symmetrical pentagonal ring with boron atoms. Using this method, a series of models were constructed, and the binding energies were calculated. Because the cluster of metal atoms is very disadvantageous to hydrogen adsorption, some models have been elected with the binding energy of each Ti atom less than the cohesive energy of Ti (−4.85 eV) [54]. Taking these thoughts into consideration, a series of non-equivalent models were constructed. As shown in Figure 1c, the binding energy for the model B123 is −4.91 eV, which is lower than the cohesive energy of Ti when the boron atoms replace the carbon atoms at position 123. Thus, C_20_ fullerene with three boron atoms doped on the C_20_ fullerene is adopted here. The most stable doped model was displayed in Figure 1b. Herein, we further analyze the stability of the adsorbent structure using an ab initio molecular dynamics simulation, as shown in Figure 1d. The simulation results show that the fluctuations for both bond length and energy are very small, demonstrating that this adsorbent structure has only tiny deformation under ambient conditions. The simulations ensure that the Ti atom will not dislocate from the carbon nanostructure at the desorption temperature and temperature below. This phenomenon is similar to the results for Ti-decorated boron-doped twin graphene, where five and six boron atoms are doped on twin graphene [55].

### 3.2. Hydrogen Adsorption

**Figure 2:**
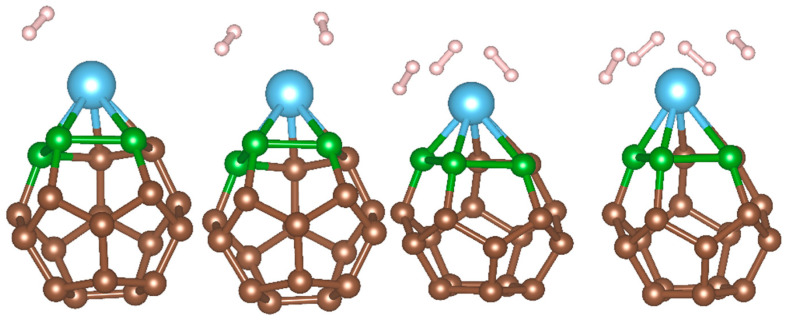
Hydrogen adsorption on the B123 model, where brown, green, blue and white represent carbon, boron, titanium and hydrogen atoms respectively.

After selecting and examining the adsorbent structure, we continue to build hydrogen adsorption models, as shown in Figure 2. The number of hydrogen molecules increases step by step in the order of left to right. This result is consistent with previous studies where each Ti atom could adsorb up to four H_2_ molecules, regarding the standard performance of one Ti atom [51,52,53,56]. For $n_{H_{2}}$ = 5, $\Delta E_{ads}$ is found to be the near-zero value of −0.055 eV, which means that H_2_ adsorption has already reached the saturated adsorption state with four H_2_ molecules adsorbed. We calculated the adsorption energy ($E_{ad}$) and the consecutive adsorption energy ($E_{cad}$) of these four models. The results clearly show that four hydrogen molecules are strongly adsorbed. The average adsorption energy is −1.52 eV, and the peak of adsorption energy is −2.36 eV. Slight Jahn−Teller distortion can be observed; the nature of a Jahn−Teller distortion could be manipulated by the application of pressure or temperature [54]. These results also prove the stability of the hydrogen adsorption. 

### 3.3. Calculations of Desorption Temperature and Gravimetric Weight Percentage of Hydrogen

**Figure 3:**
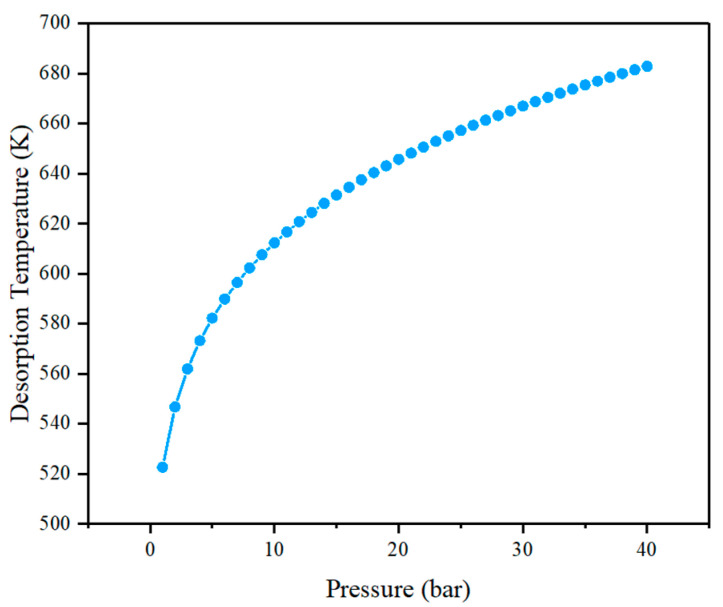
Desorption temperature as a function of pressure in the B123 model.

Desorption temperature is a vital datum that measures both the stability of a hydrogen adsorption and if it can be put into practical use. We calculated the average desorption temperature of hydrogen molecules using the Van’t Hoff equation [55].

(4) $$\begin{array}{c} T_{d}=\left(\frac{\bar{E_{cad}}}{k}\right){\left(\frac{\Delta S}{R}-lnP\right)}^{-1} \end{array}$$

where $T_{d}$ is the desorption temperature of the hydrogen molecules, $\bar{E_{cad}}$ is the average consecutive adsorption energy of −0.708 eV, ΔS is the entropy difference of hydrogen in transition from the gaseous to the liquid state, R is the gas constant, and P is the pressure. Using this method, we found that the average desorption temperature is 522.5 K under standard atmospheric pressure. The $T_{d}$ under different pressures was calculated, as shown in Figure 3. When the pressure of a gas container is 12 bar, the desorption temperature is 616.7 K. It can reach 682.8 K when the pressure rises to 40 bar. We can notice that the stable conditions of this titanium-decorated boron-doped C_20_ fullerene under ambient temperature and pressure are mild, indicating carbon-based materials have application prospects in the field of hydrogen storage.

Gravimetric weight percentage indicates the capability of hydrogen molecules, and it is also a very important parameter for hydrogen storage materials. Gravimetric weight percentage is calculated by the equation below:

(5) $$\begin{array}{c} GWP=\frac{g_{absorbent\;structure}}{g_{system}}\times 100\% \end{array}$$

where $g_{absorbent\;structure}$ is the weight of the absorbent structure, and $g_{system}$ is the weight of the absorbent structure with all hydrogen molecules absorbed. On the basis of the above analysis, one Ti atom can adsorb up to four hydrogen molecules stably. If we put the other Ti atom on top of the symmetrical pentagonal carbon ring, there are eight hydrogen molecules adsorbed on the C_20_ substrate, and a gravimetric capacity of 4.68% can be achieved. 

### 3.4. Thermomechanical Analysis

As a result of serving under ambient conditions, we studied the stability of this material under different temperatures and pressures. We used relative energy to measure its stability [57,58,59], which is given by the following:

(6) $$\begin{array}{c} E_{r}=E_{ad}-n\mu_{H_{2}}\left(T,P\right) \end{array}$$

where n is the number of absorbed hydrogen molecules, and $\mu_{H_{2}}\left(T,P\right)$ is the chemical potential of hydrogen under temperature *T* and pressure *P*. $\mu_{H_{2}}\left(T,P\right)$ is calculated from [60] the following:

(7) $$\begin{array}{c} \mu_{H_{2}}\left(T,P\right)=H\left(T\right)-H\left(0\right)-T\left[S\left(T\right)-S\left(0\right)\right]+kTln\left(\frac{P}{P_{0}}\right) \end{array}$$

where k is the Boltzmann constant, H(T) and H(0) represent the enthalpy of hydrogen at temperature *T* and 0 K, respectively, S(T) and S(0) represent the entropy of hydrogen at temperature *T* and 0 K, respectively, and $P_{0}$ is the standard atmospheric pressure of 0.1 MPa. The data needed in Equation (7) are obtained from the NIST database [61].

**Figure 4:**
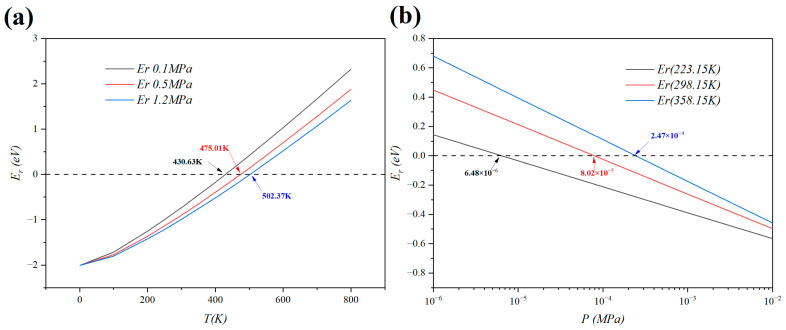
(**a**) Relative energy as a function of temperature under a given pressure in the B123 model. (**b**) Relative energy as a function of pressure under a given temperature in the B123 model.

According to Equation (6), an adsorption system is stable when $E_{r}$ is negative. We calculated $E_{r}$ under three different pressure conditions to simulate ambient serving conditions. The three reference pressure conditions are given by the U.S. Department of Energy. From Figure 4a, it can be clearly concluded that a hydrogen adsorption system requires higher pressure and lower temperature to maintain better stability. This adsorption system is stable at 430.63 K, 475.01 K, and 502.37 K under 0.1 MPa, 0.5 MPa, and 1.2 MPa, respectively. All three figures are far higher than room temperature and outdoor temperature, indicating that this adsorption system can be used under common serving conditions and can bear high temperatures since it reaches over 200 °C. Also, as shown in Figure 4b, the system can remain stable under very low pressures of 6.48 × 10^−6^ MPa at 223.15 K, 8.02 × 10^−5^ MPa at 298.15 K, and 2.47 × 10^−4^ MPa at 358.15 K. These data indicate that there is no need for a large increase in pressure to balance the temperature variation within the range of service. A thermomechanical analysis shows that this adsorption system is very stable under the given serving conditions, and this indicates a bright and promising prospect for the commercial applications of this hydrogen storage material. The calculation results mentioned above are valued and convincing because this calculation method has been validated by the works of peers with experiments [62,63].

### 3.5. Bonding Mechanism and Orbital Interactions between Titanium Atoms and C_20_ Fullerene

#### 3.5.1. Total Density of States (TDOS) Analysis

**Figure 5:**
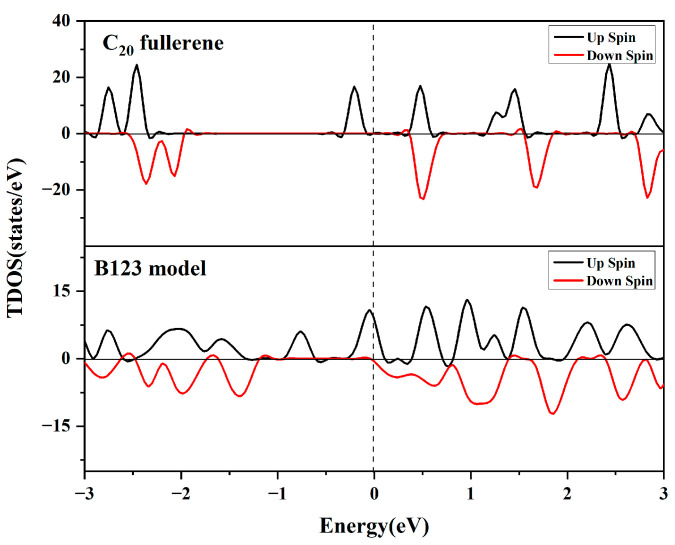
Total density of states of C_20_ fullerene and B123 model.

**Figure 6:**
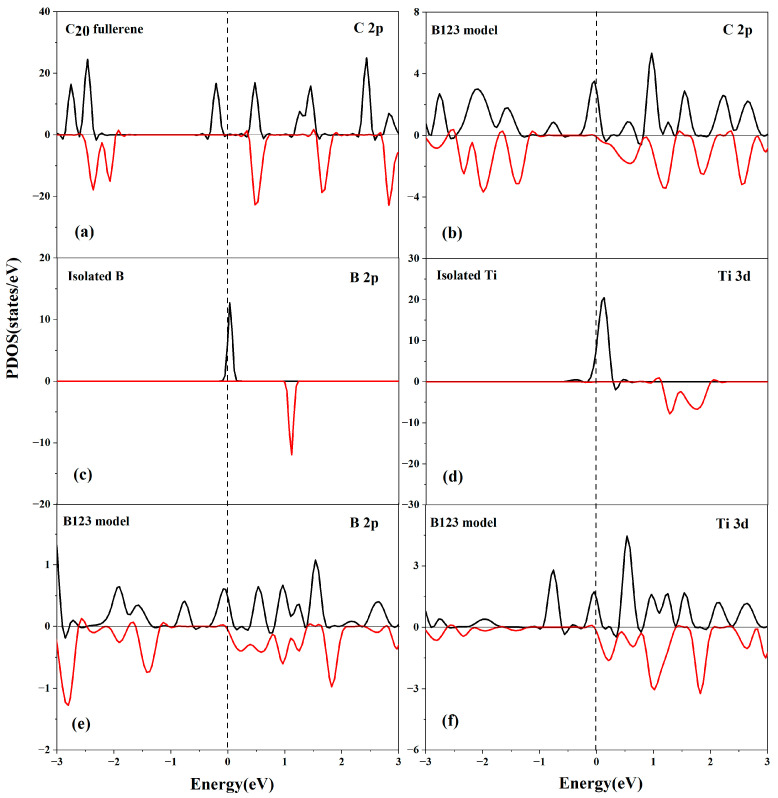
Partial density of states for (**a**) C 2p orbital of C_20_; (**b**) C 2p orbital of B123; (**c**) B 2p orbital of isolated B; (**d**) Ti 3d orbital of isolated Ti; (**e**) B 2p orbital of B123; and (**f**) Ti 3d orbital of B123.

To elucidate the origin of the dopant dependence of B/Ti adsorption energy, the spin-polarized total density of states (TDOS) of C_20_ fullerene and the B123 model are studied, as shown in Figure 5. The primary contributions to the TDOS of C_20_ fullerene stem from the C 2p orbitals, corresponding to Figure 6a. The band gap of C_20_ fullerene was found to be 0.47 eV, while there is no band gap after being doped with B and Ti.

#### 3.5.2. Partial Density of States (PDOS) Analysis

As shown in Figure 6, the C 2p orbitals, B 2p orbitals, and Ti 3d orbitals significantly contribute to the total DOS of the B123 system. The presence of B 2p peaks and Ti 3d peaks at the Fermi level further signifies the orbital hybridization between the C atom, B atom, and Ti atom, as expected. This electronic interaction leads to the formation of bonds between the C atom, B atom, and the metals, resulting in the very high cohesive energy of the Ti atom. This suggests obvious electron transfer between the Ti atom and C_20_ fullerene in the presence of a 3B atom.

### 3.6. Bonding Mechanism and Orbital Interactions between H_2_ Molecules and Ti-Decorated B-Doped C_20_ Fullerene

#### 3.6.1. Partial Density of States (PDOS) Analysis after H_2_ Adsorption

**Figure 7:**
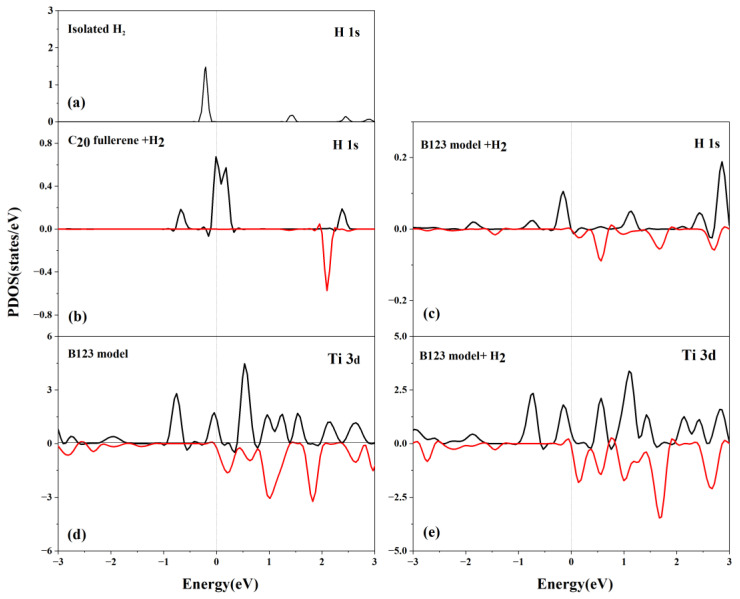
Partial density of states for the (**a**) H 1s orbital of isolated H_2_; (**b**) H 1s orbital of C_20_ fullerene + H_2_; (**c**) H 1s orbital of B123 + H_2_; (**d**) Ti 3d orbital of B123; and (**e**) Ti 3d orbital of B123 + H_2_. Fermi level is set to zero energy.

To clarify the interaction of H_2_ with C_20_, the PDOS for the H 1s orbital of isolated H_2_ and H_2_-adsorbed C_20_ fullerene and the Ti 3d orbital of the H_2_-adsorbed B123 system is shown in Figure 7. The 1s orbital of the H atom in the isolated H_2_ has a peak at the Fermi level, which is highly localized and occupied. For the adsorbed H_2_, there is a visible upshift in the 1s orbital of the H atom in the C_20_ + H_2_ system. The upshift leads to a more-filled H 1s orbital, which typically leads to weaker binding. For the B123 + H_2_ system, the 1s orbital of the H atom becomes delocalized, suggesting strong interaction between the H and the Ti atoms. The hybridization of the B 2p orbitals and Ti 3d orbitals plays a beneficial role in the high activity of the B123 toward adsorbing H_2_.

#### 3.6.2. Bader Charge Analysis

The interaction between the H_2_ molecule and C_20_ fullerene has been characterized by a significant charge transfer phenomenon. Notably, 0.4 e of the H_2_ molecule is transferred to the C_20_ fullerene.

In contrast, when the B123 system interacts with H_2_, a reverse charge transfer process is observed. Specifically, 0.11 e of charge is transferred from the B123 system to H_2_. The electron flow from the fullerene system to the H_2_ molecule is beneficial for the adsorption of hydrogen. This charge transfer process is accompanied by a redistribution of electrons within the B123 system. The Ti atom lost 1.28 e while the B and C atoms gained 0.83 e and 0.34 e, respectively.

#### 3.6.3. Charge Density Difference Analysis

**Figure 8:**
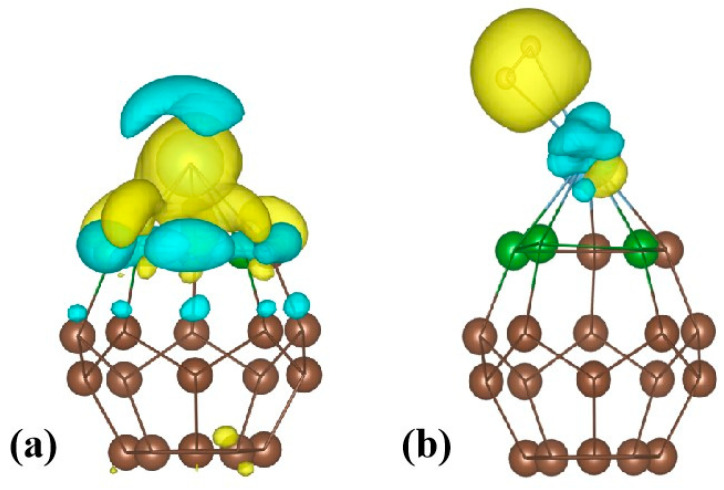
Charge density difference for the (**a**) B123 system; (**b**) B123 + H_2_ system. Yellow and blue colors represent charge-gained and charge-lost regions, respectively.

To gain a deeper understanding of the charge transfer phenomenon, we employed a visualization approach by plotting the charge density difference, as shown in Figure 8. Figure 8a reveals that a significant amount of charge is transferred from the Ti atom to the B and C atoms of C_20_ fullerene. This electron transfer is evidenced by a notable decrease in charge density around the Ti atom and a corresponding increase for the B and C atoms of C_20_ fullerene.

As presented in Figure 8b, in addition to the charge transfer observed within C_20_ fullerene, there is a further transfer of charge from the Ti atom to the H atoms of the H_2_ molecule. This manifests as an enhancement in charge density around the H atoms, indicating a strengthened Ti-H bond due to the electrons transferred from the Ti atom. This observation is consistent with the results obtained from the Bader charge analysis and partial density of states analysis.

## 4. Conclusions

On the basis of the first-principle calculations, the hydrogen storage in titanium-decorated boron-doped C_20_ fullerenes has been investigated. It is an essential method for decorated Ti on C_20_ fullerene to improve the hydrogen storage capacity. A series of non-equivalent structures by doping position and amount of boron atoms are established, and the binding energy of each Ti atom is calculated. When the 1, 2, and 3 carbon atoms on the pentagonal ring are replaced by boron atoms, this structure can adsorb four hydrogen molecules with a gravimetric weight percentage of hydrogen of 4.68%. From thermodynamic calculations, this adsorption system is stable at 430.63 K, 475.01 K, and 502.37 K under 0.1 MPa, 0.5 MPa, and 1.2 MPa, respectively. In order to explore the electronic structure and charge transfer mechanisms, the partial density of states and a Bader charge analysis were analyzed. The above research reveals the hydrogen storage capability of Ti-decorated boron-doped C_20_ fullerene, which will motivate experimentalists to deeply study the hydrogen storage capability and provide new inspirations for the discovery of carbon-based hydrogen storage materials.

## Data Availability

The data presented in this study are available in the article.

## References

[1] Ball M., Wietschel M. (2009). The future of hydrogen—Opportunities and challenges. Int. J. Hydrogen Energy.

[2] Midilli A., Dincer I. (2008). Hydrogen as a renewable and sustainable solution in reducing global fossil fuel consumption. Int. J. Hydrogen Energy.

[3] Zhu Q.-L., Xu Q. (2015). Liquid organic and inorganic chemical hydrides for high-capacity hydrogen storage. Energy Environ. Sci..

[4] Pukazhselvan D., Kumar V., Singh S.K. (2012). High capacity hydrogen storage: Basic aspects, new developments and milestones. Nano Energy.

[5] Niaz S., Manzoor T., Pandith A.H. (2015). Hydrogen storage: Materials, methods and perspectives. Renew. Sustain. Energy Rev..

[6] Li C., Yang W., Liu H., Liu X., Xing X., Gao Z., Dong S., Li H. (2024). Picturing the Gap Between the Performance and US-DOE’s Hydrogen Storage Target: A Data-Driven Model for MgH_2_ Dehydrogenation. Angew. Chem. Int. Ed..

[7] Xu W., Takahashi K., Matsuo Y., Hattori Y., Kumagai M., Ishiyama S., Kaneko K., Iijima S. (2007). Investigation of hydrogen storage capacity of various carbon materials. Int. J. Hydrogen Energy.

[8] Lin H.-J., Lu Y.-S., Zhang L.-T., Liu H.-Z., Edalati K., Révész Á. (2022). Recent advances in metastable alloys for hydrogen storage: A review. Rare Met..

[9] Dong S., Li C., Wang J., Liu H., Ding Z., Gao Z., Yang W., Lv W., Wei L., Wu Y. (2022). The “burst effect” of hydrogen desorption in MgH_2_ dehydrogenation. J. Mater. Chem. A.

[10] Dong S., Li C., Lv E., Wang J., Liu H., Gao Z., Xiong W., Ding Z., Yang W., Li H. (2022). MgH_2_/single-atom heterojunctions: Effective hydrogen storage materials with facile dehydrogenation. J. Mater. Chem. A.

[11] Norberg N.S., Arthur T.S., Fredrick S.J., Prieto A.L. (2011). Size-Dependent Hydrogen Storage Properties of Mg Nanocrystals Prepared from Solution. J. Am. Chem. Soc..

[12] Chung K.-H. (2010). High-pressure hydrogen storage on microporous zeolites with varying pore properties. Energy.

[13] Ren J., Musyoka N.M., Annamalai P., Langmi H.W., North B.C., Mathe M. (2015). Electrospun MOF nanofibers as hydrogen storage media. Int. J. Hydrogen Energy.

[14] Kappes M., Iannuzzi M., Carranza R.M. (2013). Hydrogen Embrittlement of Magnesium and Magnesium Alloys: A Review. J. Electrochem. Soc..

[15] Sakintuna B., Lamaridarkrim F., Hirscher M. (2007). Metal hydride materials for solid hydrogen storage: A review. Int. J. Hydrogen Energy.

[16] Schlapbach L., Züttel A. (2001). Hydrogen-storage materials for mobile applications. Nature.

[17] Bhatia P., Chandra R., Nath M. (2024). Controlled synthesis of ZIF-11 with varied particle size: Effective adsorbent for industrial pollutants and host for storage of gaseous CO_2_, H_2_ and CH_4_. Mater Chem Phys.

[18] Szczesniak B., Choma J., Jaroniec M. (2018). Gas adsorption properties of hybrid graphene-MOF materials. J. Colloid Interface Sci..

[19] Zollo G., Gala F. (2012). Atomistic Modeling of Gas Adsorption in Nanocarbons. J. Nanomater..

[20] Salehabadi A., Umar M.F., Ahmad A., Ahmad M.I., Ismail N., Rafatullah M. (2020). Carbon-based nanocomposites in solid-state hydrogen storage technology: An overview. Int. J. Energy Res..

[21] Siddiqui M.T.H., Nizamuddin S., Baloch H.A., Mubarak N.M., Al-Ali M., Mazari S.A., Bhutto A.W., Abro R., Srinivasan M., Griffin G. (2019). Fabrication of advance magnetic carbon nano-materials and their potential applications: A review. J. Environ. Chem. Eng..

[22] Pyle D.S., Gray E.M., Webb C.J. (2016). Hydrogen storage in carbon nanostructures via spillover. Int. J. Hydrogen Energy.

[23] Park Y.-J., Lee H., Choi H.L., Tapia M.C., Chuah C.Y., Bae T.-H. (2023). Mixed-dimensional nanocomposites based on 2D materials for hydrogen storage and CO_2_ capture. npj 2d Mater. Appl..

[24] Nagar R., Vinayan B.P., Samantaray S.S., Ramaprabhu S. (2017). Recent advances in hydrogen storage using catalytically and chemically modified graphene nanocomposites. J. Mater. Chem. A.

[25] Duan Z., Shi S., Yao C., Liu X., Diao K., Lei D., Liu Y. (2024). Reversible hydrogen storage with Na-modified Irida-Graphene: A density functional theory study. Int. J. Hydrogen Energy.

[26] Zhou M., Lu Y., Zhang C., Feng Y.P. (2010). Strain effects on hydrogen storage capability of metal-decorated graphene: A first-principles study. Appl. Phys. Lett..

[27] Zafar M., Iqbal T., Fatima S., Sanaullah Q., Aman S. (2021). Carbon nanotubes for production and storage of hydrogen: Challenges and development. Chem. Pap..

[28] Verma R., Jaggi N. (2024). Ability of transition metal and hetero atoms co-doped SWCNTs for hydrogen adsorption: A DFT study. J. Phys. Chem. Solids.

[29] Joseph J., Sivasankarapillai V.S., Nikazar S., Shanawaz M.S., Rahdar A., Lin H., Kyzas G.Z. (2020). Borophene and Boron Fullerene Materials in Hydrogen Storage: Opportunities and Challenges. ChemSusChem.

[30] Ma L.-P., Wu Z.-S., Li J., Wu E.-D., Ren W.-C., Cheng H.-M. (2009). Hydrogen adsorption behavior of graphene above critical temperature. Int. J. Hydrogen Energy.

[31] Züttel A. (2003). Materials for hydrogen storage. Mater. Today.

[32] Verma R., Jaggi N. (2024). A DFT investigation of Osmium decorated single walled carbon nanotubes for hydrogen storage. Int. J. Hydrogen Energy.

[33] Zhang H., Zhao X., Zhang M., Luo Y., Li G., Zhao M. (2013). Three-dimensional diffusion of molecular hydrogen in graphdiyne: A first-principles study. J. Phys. D Appl. Phys..

[34] Dai W., Xiao M., Chen M.-Q., Xu J.-J., Tang Y.-J. (2016). A simulation study on the hydrogen storage properties of fullerene family molecules Cx (x = 56, 60, 70) and their hydrides. Mod. Phys. Lett. B.

[35] Paul D., Mane P., Sarkar U., Chakraborty B. (2023). Yttrium decorated fullerene C_30_ as potential hydrogen storage material: Perspectives from DFT simulations. Theor. Chem. Acc..

[36] Tang C., Chen S., Zhu W., Zhang A., Zhang K., Zou H. (2014). Doping the transition metal atom Fe, Co, Ni into C_48_B_12_ fullerene for enhancing H_2_ capture: A theoretical study. Int. J. Hydrogen Energy.

[37] Mahamiya V., Shukla A., Chakraborty B. (2022). Exploring yttrium doped C_24_ fullerene as a high-capacity reversible hydrogen storage material: DFT investigations. J. Alloys Compd..

[38] Huang W., Shi M., Song H., Wu Q., Huang X., Bi L., Yang Z., Wang Y. (2020). Hydrogen storage on chains-terminated fullerene C_20_ with density functional theory. Chem. Phys. Lett..

[39] Muniyandi S., Sundaram R., Kar T. (2023). A comparison study on the sensing ability of C_20_/B_12_N_12_ nanocage towards beryllium hydride cluster and beryllium hydride molecules using density functional theory (DFT). Mater. Today Commun..

[40] Ammar H.Y., Badran H.M. (2021). Ti deposited C_20_ and Si_20_ fullerenes for hydrogen storage application, DFT study. Int. J. Hydrogen Energy.

[41] Kareem R.T., Ahmadi S., Rahmani Z., Ebadi A.G., Ebrahimiasl S. (2021). Characterization of titanium influences on structure and thermodynamic stability of novel C_20-n_Ti_n_ nanofullerenes (n = 1–5): A density functional perspective. J. Mol. Model..

[42] Sun Q., Wang Q., Jena P., Kawazoe Y. (2005). Clustering of Ti on a C_60_ surface and its effect on hydrogen storage. J. Am. Chem. Soc..

[43] Zhang H., Tong C.-J., Zhang Y., Zhang Y.-N., Liu L.-M. (2015). Porous BN for hydrogen generation and storage. J. Mater. Chem. A.

[44] Kim D., Lee S., Jo S., Chung Y.-C. (2013). Strain effects on hydrogen storage in Ti decorated pyridinic N-doped graphene. Phys. Chem. Chem. Phys..

[45] Parkar P., Chaudhari A. (2024). Hydrogen storage properties of Ti-doped C20 nanocage and its derivatives: A comprehensive density functional theory investigation. Mater. Chem. Phys..

[46] Kresse G., Hafner J. (1993). Ab initio molecular dynamics for open-shell transition metals. Phys. Rev. B Condens. Matter.

[47] Ernzerhof M., Scuseria G.E. (1999). Assessment of the Perdew–Burke–Ernzerhof exchange-correlation functional. J. Chem. Phys..

[48] Perdew J.P., Burke K., Ernzerhof M. (1996). Generalized Gradient Approximation Made Simple. Phys. Rev. Lett..

[49] Grimme S., Antony J., Ehrlich S., Krieg H. (2010). A consistent and accurate ab initio parametrization of density functional dispersion correction (DFT-D) for the 94 elements H-Pu. J. Chem. Phys..

[50] Grimme S., Ehrlich S., Goerigk L. (2011). Effect of the damping function in dispersion corrected density functional theory. J. Comput. Chem..

[51] Pan H.-Z., Wang Y.-L., He K.-H., Wei M.-Z., Ouyang Y., Chen L. (2013). First-principles study of hydrogen adsorption on titanium-decorated single-layer and bilayer graphenes. Chin. Phys. B.

[52] Ma K., Lv E., Zheng D., Cui W., Dong S., Yang W., Gao Z., Zhou Y. (2021). A First-Principles Study on Titanium-Decorated Adsorbent for Hydrogen Storage. Energies.

[53] Faye O., Szpunar J.A. (2018). An Efficient Way To Suppress the Competition between Adsorption of H_2_ and Desorption of nH_2_-Nb Complex from Graphene Sheet: A Promising Approach to H_2_ Storage. J. Phys. Chem. C.

[54] Slanina Z., Adamowicz L. (1993). One-, Two- and Three-Dimensional Structures of C20. Fuller. Sci. Technol..

[55] Si L., Tang C. (2017). The reversible hydrogen storage abilities of metal Na (Li, K, Ca, Mg, Sc, Ti, Y) decorated all-boron cage B28. Int. J. Hydrogen Energy.

[56] Meng T., Wang C.-Y., Wang S.-Y. (2007). First-principles study of a single Ti atom adsorbed on silicon carbide nanotubes and the corresponding adsorption of hydrogen molecules to the Ti atom. Chem. Phys. Lett..

[57] Chen X., Wang L., Zhang W., Zhang J., Yuan Y. (2017). Ca-decorated borophene as potential candidates for hydrogen storage: A first-principle study. Int. J. Hydrogen Energy.

[58] Wang J., Du Y., Sun L. (2016). Ca-decorated novel boron sheet: A potential hydrogen storage medium. Int. J. Hydrogen Energy.

[59] Wang L., Chen X., Du H., Yuan Y., Qu H., Zou M. (2018). First-principles investigation on hydrogen storage performance of Li, Na and K decorated borophene. Appl. Surf. Sci..

[60] Dong S., Lv E., Wang J., Li C., Ma K., Gao Z., Yang W., Ding Z., Wu C., Gates I.D. (2021). Construction of transition metal-decorated boron doped twin-graphene for hydrogen storage: A theoretical prediction. Fuel.

[61] Chase M.W. (1998). NIST-JANAF Thermochemical Tables.

[62] Gao Y., Li Z., Wang P., Cui W.G., Wang X., Yang Y., Gao F., Zhang M., Gan J., Li C. (2024). Experimentally validated design principles of heteroatom-doped-graphene-supported calcium single-atom materials for non-dissociative chemisorption solid-state hydrogen storage. Nat. Commun..

[63] García-Arroyo E., Reider A.M., Kollotzek S., Foitzik F., Campos-Martínez J., Bartolomei M., Pirani F., Hernández M.I., Mella M., Scheier P. (2024). The role of Na decoration on the hydrogen adsorption on coronene: A combined experimental and computational study. Int. J. Hydrogen Energy.

